# Breaking Down Barriers and Paving the Way for Blockchain Adoption in Dentistry

**DOI:** 10.7759/cureus.39166

**Published:** 2023-05-17

**Authors:** Raheel Allana, Asad Allana, Syed Sarosh Mahdi

**Affiliations:** 1 Paediatrics & Child Health/Epidemiology and Biostatistics, Aga Khan University, Karachi, PAK; 2 Community Health Sciences/Epidemiology and Biostatistics, Aga Khan University, Karachi, PAK; 3 Dentistry, International Medical University, Kuala Lumpur, MYS

**Keywords:** clinical data management, dental clinic, barriers, digital dentistry, blockchain

## Abstract

Blockchain technology can potentially transform the dentistry sector by facilitating safe communication between dental practitioners and offering secure and efficient administration of patient information. Nevertheless, using this technology in dentistry confronts various barriers, including regulatory and legal obstacles, a lack of technical skills, and a lack of standardization. To overcome these challenges, dental practitioners, industry stakeholders, and regulators must work together to develop a legislative framework that encourages the use of blockchain technology in dentistry. Moreover, education and training programs must equip dental practitioners with the skills and expertise to properly incorporate and use blockchain technology. The use of blockchain technology in dentistry has the potential to greatly improve patient outcomes while also increasing the efficiency and security of the dental business.

## Editorial

Technology has significantly improved healthcare outcomes, optimized healthcare delivery, and increased access to healthcare services [1]. Blockchain technology is one of the most recent technological advances that could revolutionize the healthcare sector [2]. This decentralized and secure digital ledger can potentially improve data security, patient confidentiality, and transparency of healthcare transactions. Like other healthcare sectors, dentistry can benefit from incorporating blockchain technology into its operations [3].

Integrating blockchain technology in dentistry has the potential to alter the way dental offices run by leveraging the blockchain's security, transparency, and data integrity. Blockchain technology, by establishing a decentralized database of patient information, can give an unparalleled degree of security that is resistant to hacking and cyber threats [4]. It can also allow dental clinics to communicate patient information in real-time, allowing for greater care coordination, more accurate diagnoses, and better treatment options. This can eventually lead to improved patient outcomes and satisfaction. Additionally, blockchain technology can potentially eliminate inefficiencies in dental clinics' invoicing and insurance systems. Blockchain can lower the risk of fraud, simplify claim handling, and enable speedier payouts by building a tamper-proof and transparent payment system [5]. This can result in cost reductions for dental offices and patients and an improvement in the dentistry industry's overall financial health.

Furthermore, patient records can be securely maintained using blockchain technology, and access can be provided only to authorized personnel, protecting the confidentiality and integrity of critical information [3]. Using blockchain technology in dentistry might thus lead to a more efficient, secure, and patient-centered dental care system, increasing patients' trust and confidence in the dental business. While little information is available on the names of software systems designed expressly for dentistry, blockchain platforms such as Ethereum, Hyperledger Fabric, and Corda may be utilized to construct custom solutions for patient data management. These platforms provide the infrastructure and tools to develop blockchain-powered decentralized apps (DApps). These platforms are assessed by modifying the deployed concurrent transactions as workloads and increasing network size (nodes) to determine the scalability [5].

However, while blockchain technology has significant advantages in dentistry, some impediments to its adoption exist:

Dental clinics face inconsistent software systems that hinder safe patient data transfer. Blockchain can provide a decentralized solution, but standards and cooperation are necessary for effective implementation.

Additionally, regulatory and legal constraints may hinder blockchain technology's adoption in dentistry. The dentistry profession is heavily regulated, with tight requirements for managing and storing patient data. Because blockchain technology is still in its early phases, there may be questions about how it will comply with existing rules such as HIPAA (Health Insurance Portability and Accountability Act). This may present legal impediments to blockchain technology adoption in the dental industry, as dental clinics may be unwilling to employ a system that does not conform with existing regulations.

Another limitation of blockchain is its scalability, transaction, and latency assessment. The scalability and transactional latency tests are essential because it would be difficult for the blockchain and all participating nodes to function if a large number of nodes/peers (dental clinics) were added to the blockchain network at the same time and sent data/transactions that scaled to a larger size.

Lastly, the legislative and legal environment governing the use of blockchain technology in dentistry is still growing. The absence of clear standards and restrictions on how blockchain may be utilized in healthcare may cause dental practitioners to hesitate to use the technology. The inherent privacy and security problems connected with blockchain use in healthcare may potentially impede its adoption. To overcome these obstacles, dental stakeholders will need to collaborate and educate one another and set rules and laws to ensure the safe and successful use of blockchain technology in dentistry.

Overall, Blockchain technology can potentially transform the dentistry profession, but various challenges must be solved before it can be widely used. Educating dental practitioners about the benefits of blockchain technology and its possible uses is critical. It is also critical to make blockchain technologies more inexpensive and accessible to dental clinics. The legislative and legal framework surrounding blockchain technology in healthcare must also be built to assure patient and practitioner safety and security. The dentistry profession can reap the benefits of a more secure, transparent, and efficient system by working together to tackle these difficulties.
